# KIF20A Regulates Porcine Oocyte Maturation and Early Embryo Development

**DOI:** 10.1371/journal.pone.0102898

**Published:** 2014-07-18

**Authors:** Yu Zhang, Jun Liu, Xu Peng, Cheng-Cheng Zhu, Jun Han, Jia Luo, Rong Rui

**Affiliations:** 1 College of Veterinary Medicine, Nanjing Agricultural University, Jiangsu, China; 2 College of Animal Sciences and Technology, Nanjing Agricultural University, Jiangsu, China; 3 Technology Centre of Guangxi Entry-Exit Inspection and Quarantine Bureau, Nanning, China; South China Agricultural University, China

## Abstract

KIF20A (Kinesin-like family member 20A), also called mitotic kinesin-like proteins 2 (MKLP2), is a mammalian mitotic kinesin-like motor protein of the Kinesin superfamily proteins (KIFs), which was originally involved in Golgi apparatus dynamics and thought to essential for cell cycle regulation during successful cytokinesis. In the present study, we investigated whether KIF20A has roles on porcine oocyte meiotic maturation and subsequent early embryo development. By immunofluorescence staining, KIF20A was found to exhibit a dynamic localization pattern during meiosis. KIF20A was restricted to centromeres after germinal vesicle breakdown (GVBD), transferred to the midbody at telophase I (TI), and again associated with centromeres at metaphase II (MII). Inhibition of endogenous KIF20A via a specific inhibitor, Paprotrain, resulted in failure of polar body extrusion. Further cell cycle analysis showed that the percentage of oocytes that arrested at early metaphase I (MI) stage increased after KIF20A activity inhibition; however, the proportion of oocytes at anaphase/telophase I (ATI) and MII stages decreased significantly. Our results also showed that KIF20A inhibition did not affect spindle morphology. In addition, KIF20A was localized at the nucleus of early embryos, and KIF20A inhibition resulted in failure of early parthenogenetic embryo development. These results demonstrated that KIF20A is critical for porcine oocyte meiotic maturation and subsequent early embryo development.

## Introduction

To maintain genomic stability, normal multiplication of animal cell must undergo replication of the genome, chromosome segregation and subsequent cytokinesis. In mitosis, cytokinesis is achieved by separating of sister chromatids to ensure faithful segregation of the chromosomal content [1]. During meiosis, separation of homologous chromosomes and sister chromatids take place to ensure the production of haploid gametes. After chromosomal separation, the oocyte divides to form a highly polarized large MII arrested oocyte with a tiny first polar body. This process is essential for retention of maternal components for early embryo development [2].

Molecular aspects of the orderly reduction of chromosomes are still unclear. In mammalian cells, mitosis begins with the separation of centrosomes at prophase to ensure accurate chromosomal movement and segregation [3], [4]. Moreover, the central spindle, formed between segregating chromosomes during anaphase, is essential for alignment and accurate segregation of chromosomes. Furthermore, many proteins moving along the microtubules are necessary for formation of the spindle, including the mitotic kinesin and mitotic kinesin-like proteins (MKLPs) superfamily that move along the microtubules as cargo [5], [6]. A subset of motor proteins from this family, which is known as mitotic or chromosomal kinesins, is involved in spindle assembly and chromosomal segregation, which are essential for cytokinesis [7], [8]. Recently, results from several studies showed that MKLPs associated with the central spindle are required for proper cytokinesis [9]–[11].

Kinesins constitute a large family of proteins that binds to microtubules and couples ATP hydrolysis for generating mechanical force [12]. Kinesin superfamily proteins (KIFs) are known to play a prominent role in cellular functions during mitotic, such as mitotic spindle formation, chromosomal segregation and intracellular movements of organelles and vesicles [12], [13]. The kinesin 6 proteins family, a new member of the kinesin superfamily, includes mitotic kinesin-like proteins 1 (MKLP1) and mitotic kinesin-like proteins 2 (MKLP2, KIF20A), which are believed to interact with microtubules [14] and are required for mitotic progression [15]. Although belonging to the same family of kinesin 6 proteins family, they probably have their own unique functions.

KIF20A was first identified to contribute to Golgi apparatus dynamics via interaction with the GTP-bound form of Rab6 during interphase [16]. As a microtubule (MT)-based motor proteins, KIF20A was previously been reported to accumulate at midzone during anaphase I (AI), and at midbody during telophase I (TI) in mitotic cells [10], [17]. KIF20A is required during mitotic exit for the final step of cytokinesis, and depletion causes defects in cleavage furrow ingression [10], [11]. KIF20A is necessary for the localization of polo-like kinase 1 (PLK1) to the central spindle in ATI cells [11] and is also required for the regulation of the chromosomal passenger complex (CPC) to the central spindle during AI for completing cytokinesis [18]. Drosophila Subito, which is an ortholog of KIF20A in mammalian cells, localizes to the spindle midzone at AI and is also required for localization of Polo, INCENP and Aurora B, which are essential for cytokinesis [19]. It has previously been found that the regulation of KIF20A by Mad2 is important for mediating proper cytokinesis [20].

Although the roles of KIF20A in spindle formation and cytokinesis has been well studied in mitosis, it remains to be elusive whether KIF20A functions in porcine oocyte meiotic maturation and subsequent early embryo development. The present study was aimed to investigate the function of KIF20A on porcine meiosis and embryo development of porcine oocytes and to expand the understanding of the regulators of oocyte asymmetric division and embryo development, as to date only a few molecules have been identified as being involved in this process. Using a specific inhibitor Paprotrain, we found that KIF20A inhibition caused the failure of porcine oocytes meiotic maturation and early embryo development.

## Materials and Methods

### Ethics statement

Animals use and care were in accordance with Animal Research Institute Committee guidelines prescribed by Nanjing Agricultural University, China. Ovaries were obtained from 6 month-old Duroc gilts at the Nanjing Tianhuan Food Corporation slaughterhouse (Nanjing, China) and transported to the laboratory within 3 h after death in sterile physiological saline (0.9% natrium chloride) containing 75 mg / L of penicillin and 50 mg / L streptomycin at 30–35°C. This study was specifically approved by the Committee of Animal Research Institute, Nanjing Agricultural University, China, and permission was obtained from the Nanjing Tianhuan Food Corporation slaughterhouse to use the ovaries.

### Antibodies and chemicals

Rabbit polyclonal anti-KIF20A antibodies were from Santa Cruz (Santa Cruz, CA). Phalloidin-TRITC, Phalloidin-FITC, Lectin-FITC, and mouse polyclonal anti-α-tubulin-FITC antibody were purchased from Sigma-Aldrich (St Louis, MO, USA). Goat anti-rabbit Alexa Fluor 488 and 568 antibodies were purchased from Zhongshan Golden Bridge Biotechnology Co. Ltd (Beijing). The inhibitor Paprotrain, a cell-permeable acrylonitrile compound, was from Calbiochem (Merck KGaA, Darmstadt, Germany). The basic maturation culture medium was TCM199 (St. Louis, MO, USA). All other chemicals and reagents were purchased from Sigma-Aldrich unless otherwise stated. Porcine follicular fluid (pFF) was aspirated from 3–6 mm ovarian follicles. The fluid was collected and centrifuged at 2000 rpm for 30 min at 4°C, then stored at −20°C under sterile conditions before use.

### Oocytes isolation and in vitro culture

Ovaries were washed 3 times with fresh sterile Dulbecco's Phosphate Buffered Saline (DPBS) and were stored at 37°C. The cumulus-oocyte complexes (COCs) were collected from follicles (3–5 mm in diameter) by aspirating with a 20-gauge needle. COCs contained in the sediment were washed twice with DPBS. After washing 3 times with TCM199, COCs were cultured with fresh maturation medium TCM199 medium containing 0.1% (wt/vol) polyvinyl alcohol (PVA), 0.91 mM sodium pyruvate, 3.05 mM glucose, 10% (v / v) pFF, 0.1 mg /ml cysteine, 10 ng /ml epidermal growth factor, 10 IU/ml PMSG, 10 IU/ml hCG, 0.065 mg /ml penicillin and 0.05 mg /ml streptomycin. COCs surrounded by at least 3 layers of intact cumulus cells were chosen for further experiments. Approximately 60–70 selected COCs were cultured with 500 µl of maturation medium in 4-well dishes (NUNC; Nalge Nunc International, Roskilde, Denmark) at 38.5°C in humidified air with 5% CO_2_ for 44 h.

### Paprotrain treatment of porcine oocytes

For Paprotrain treatment, the KIF20A inhibitor Paprotrain was diluted to50 mM stock solution in dimethyl sulfoxide (DMSO) and stored at −20°C. At the beginning of each culture, the stock solution was diluted with TCM199 to reach final concentrations of 10, 20 and 50 µM. COCs or denuded oocytes (DOs) were cultured in the presence/absence of Paprotrain in vitro. The control groups were performed with pure DMSO at the same concentration. COCs were denuded of their cumulus cells by gentle pipetting with 0.1% (w/v) hyaluronidase. Oocytes with clearly extruded polar bodies were judged to be matured oocytes. After cultured for 44 h, the polar body extrusion rate of matured oocytes was observed using a microscope. Furthermore, chromosomal alignments and the cell cycle of oocytes treated with inhibitor were examined using laser scan confocal microscopy (Zeiss LSM 700 META, Germany) after culture.

### Activation of porcine oocytes in vitro

To produce embryos in vitro, electrical stimulation (EST) was applied to oocytes that were cultured without Paprotrain. After being denuded, oocytes were placed in a chamber filled with activation medium (0.3 M mannitol, 0.05 mM CaCl_2_ and 0.1 mM MgCl_2_ and 0.1% BSA) for 3 min. Ten to 15 oocytes were placed onto an electrode wire chamber and an electrical pulse of 0.63 kV/cm, 80 µs, and 1DC was employed to activate oocytes. After electrical activation, oocytes were washed 3 times and finally transferred into porcine zygote medium 3 (PZM-3) supplements with 0.4% BSA for embryo culture.

### Paprotrain treatment and evaluation of embryos

To evaluate the effect of KIF20A on embryo development, the Paprotrain stock solution was diluted with PZM-3 to reach final concentrations of 10, 20 and 50 µM. Parthenogenetic activation oocytes were cultured in PZM-3 in the presence/absence of Paprotrain in vitro. The control groups were performed with the same concentration of pure DMSO. After EST, 30–40 oocytes were cultured in 500 µl of PZM-3 medium with or without Paprotrain at 38.5°C in humidified air with 5% CO_2_. Both the control group and the treatment group were cultured continuously for evaluation of embryo developmental competence. The developmental capability of embryos was recorded at Day 1 or 2 when the almost all of embryos have reached the 2-cell or 4-cell stage. Embryos were removed for immunostaining after different hrs of culture.

### Immunofluorescent and confocal microscopy

Using immunofluorescence staining, oocytes or embryos were fixed in 4% paraformaldehyde (in PBS) for 30 min and treated with membrane permeabilization solution (1% Triton X-100 in PBS) for 8–12 h at room temperature. Then, oocytes or embryos were blocked in blocking buffer (1% BSA in PBS) for 1 h and incubated overnight at 4°C or 5 h at room temperature with rabbit polyclonal anti-KIF20A antibodies at 1∶100 dilution. After washing 3 times (2 min each) in washing buffer (0.1% Tween 20 and 0.01% Triton X-100 in PBS), oocytes or embryos were labeled with Alexa Fluor 488/568 goat-anti-rabbit IgG at 1∶100 dilution for 1 h at room temperature. For α-tubulin-FITC staining, after incubation for 2 h at room temperature, oocytes were washed 3 times. These samples were co-stained with Hoechst 33342 (10 µg/ml in PBS) for 10 min, mounted on glass slides, and then examined with a laser scan confocal microscopy. Thirty oocytes or embryos were examined for each group.

### Statistical analysis

At least 3 replicates were performed for each treatment. Results are expressed as means ± SEM's. Statistical comparisons were made by analysis of variance (ANOVA) followed by Duncan's multiple comparisons test. P<0.05 was considered to be significant.

## Results

### Intracellular localization of KIF20A during porcine oocyte meiotic maturation

**Figure 1:**
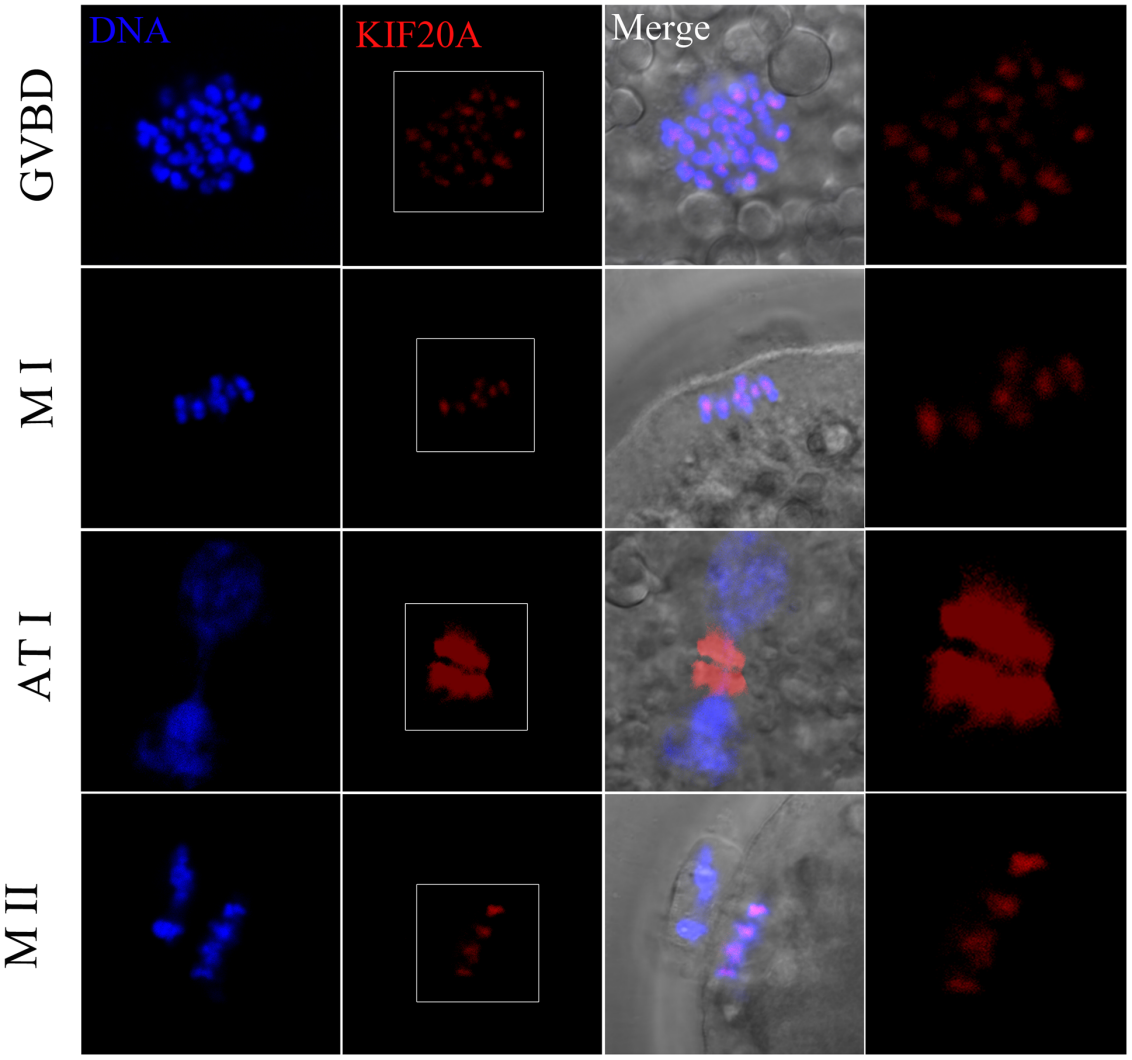
Subcellular localization of KIF20A in porcine oocytes. Immunofluorescent staining was employed to show the subcellular localization of KIF20A. KIF20A exhibited a dynamic localization pattern during porcine oocyte meiotic maturation. KIF20A localized at centromeres at the germinal vesicle breakdown (GVBD) stage and metaphase I (MI) stage, then transferred to the midbody in dividing oocytes during telophase (TI), finally, KIF20A was accumulated at centromeres again at metaphase II (MII). Red, KIF20A; blue, chromatin.

The subcellular localization of KIF20A was examined at different stages of meiotic maturation by immunofluorescent staining with a KIF20A antibody. As shown in Figure 1, KIF20A exhibited a dynamic localization pattern during meiosis. It localized at the centromeres in early MI stage, and then moved to the midbody in dividing oocytes during TI; KIF20A again accumulated at centromeres at MII.

### Inhibition of KIF20A activity results in failure of porcine oocyte maturation and cell-cycle disturbances

**Figure 2:**
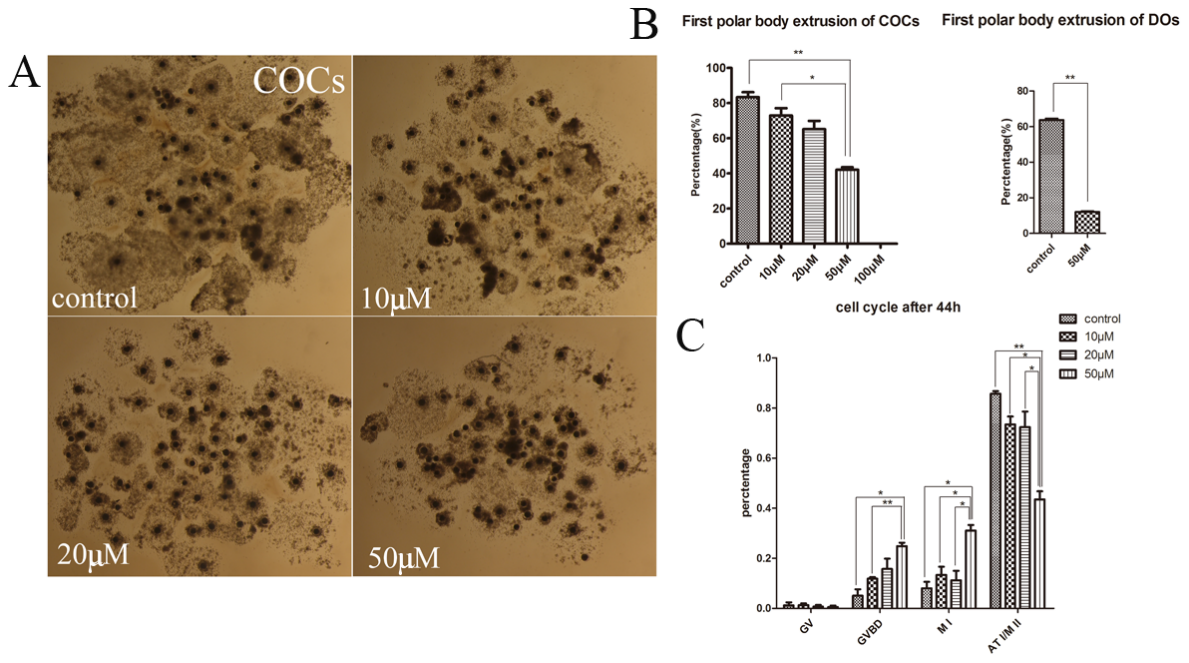
Disrupting KIF20A activity inhibits the meiosis competence and cell cycle progression of porcine oocytes. (A) Granulosa cell diffusion of COCs was inhibited during meiosis. Almost all of COCs in control groups were surrounded by more than 5 layers of intact cumulus cells, whereas the COCs in 10, 20 and 50 µM Paprotrain treatment groups showed weakened diffusion. (B) Rates of first polar body extrusion of porcine oocytes after 44 h culture with different treatments (Control, 10 µM, 20 µM, and 50 µM). Oocytes failed to extrude the first polar body after treatment with KIF20A inhibitor. * significant difference (p<0.05). (C) Effects of KIF20A disruption on cell cycle during oocyte meiosis maturation. The cell cycle progression of porcine oocytes was disturbed during meiosis. The oocytes arrested at GVBD or MI stage and cannot perform the proper progression of cell cycle after KIF20A disruption. * significant difference (p<0.05).

To inhibit endogenous KIF20A activity during meiotic maturation, porcine oocytes were treated with Paprotrain for 44 h. Figure 2A shows although almost whole of COCs in control groups were surrounded by more than 5 layers of intact cumulus cells, cumulus expansion of those COCs treated with 10, 20 and 50 µM Paprotrain became progressively poor. Furthermore, the first polar body (pbI) emission by porcine oocytes decreased in a dose-dependent manner both in COC groups and DO groups (Fig. 2B). In the control oocytes, pbI extrusion rate was 83.38±2.86%(n = 240), while it was 72.95±4.16% (n = 229) and 65.2±4.69% (n = 213) after 10 and 20 µM Paprotrain treatment respectively; however, the rate of polar body extrusion in oocytes treated with 50 µM Paprotrain was significantly reduced to 42.07±7.41% (n = 224, p<0.01).

To exclude the effects of cumulus cells on oocyte maturation, DOs were cultured with 50 µM inhibitor. Compared with controls, the percentage of oocytes with extruded pbI was sharply decreased from 63.69±0.90% (n = 91 DOs) in the control to 12.07±0.43% (n = 91 DOs; p<0.01) in the treatment groups. Thus, COCs were adopted to investigate the roles of KIF20A during porcine oocyte meiotic maturation. The results showed that the disruption of KIF20A caused the failure of polar body extrusion in porcine oocyte.

As mentioned above, Paprotrain-treated porcine oocytes suffered failure of nuclear maturation. Thus, we focused on the proportions of porcine oocytes that were arrested at different meiotic stages. After treatment with Paprotrain for 44 h, when most oocytes were supposed to reach MII stage, the meiotic stages of oocytes were assessed via immunofluorescent images of their chromosomes labeled with Hochest-33342. As shown in Figure 2C, the number of oocytes arrested at early MI stage increased in a dose-dependent manner after KIF20A activity inhibition, while the percentage of oocytes that reached ATI and MII stages decreased after treatment. In control groups, the percentages of oocytes arrested at GVBD and MI stages were 5.07±2.55% and 8.04±2.58% (n = 174), respectively, whereas that of treatment groups were significantly increased to 24.85±1.35% and 31.07±2.23% (n = 168; P<0.05) after 50 µM Paprotrain treatment. Furthermore, in comparison with the control groups in which 85.70±1.05% (n = 174) of oocytes succeeded in reaching the ATI/MII stages, 43.51±3.26% of oocytes reached ATI/MII stages in treatment groups (P<0.01) (Fig. 2C). Therefore, inhibition of KIF20A activity blocked the cell cycle progression of porcine oocyte meiotic maturation.

### KIF20A inhibition has no effect on spindle formation of porcine oocytes

**Figure 3:**
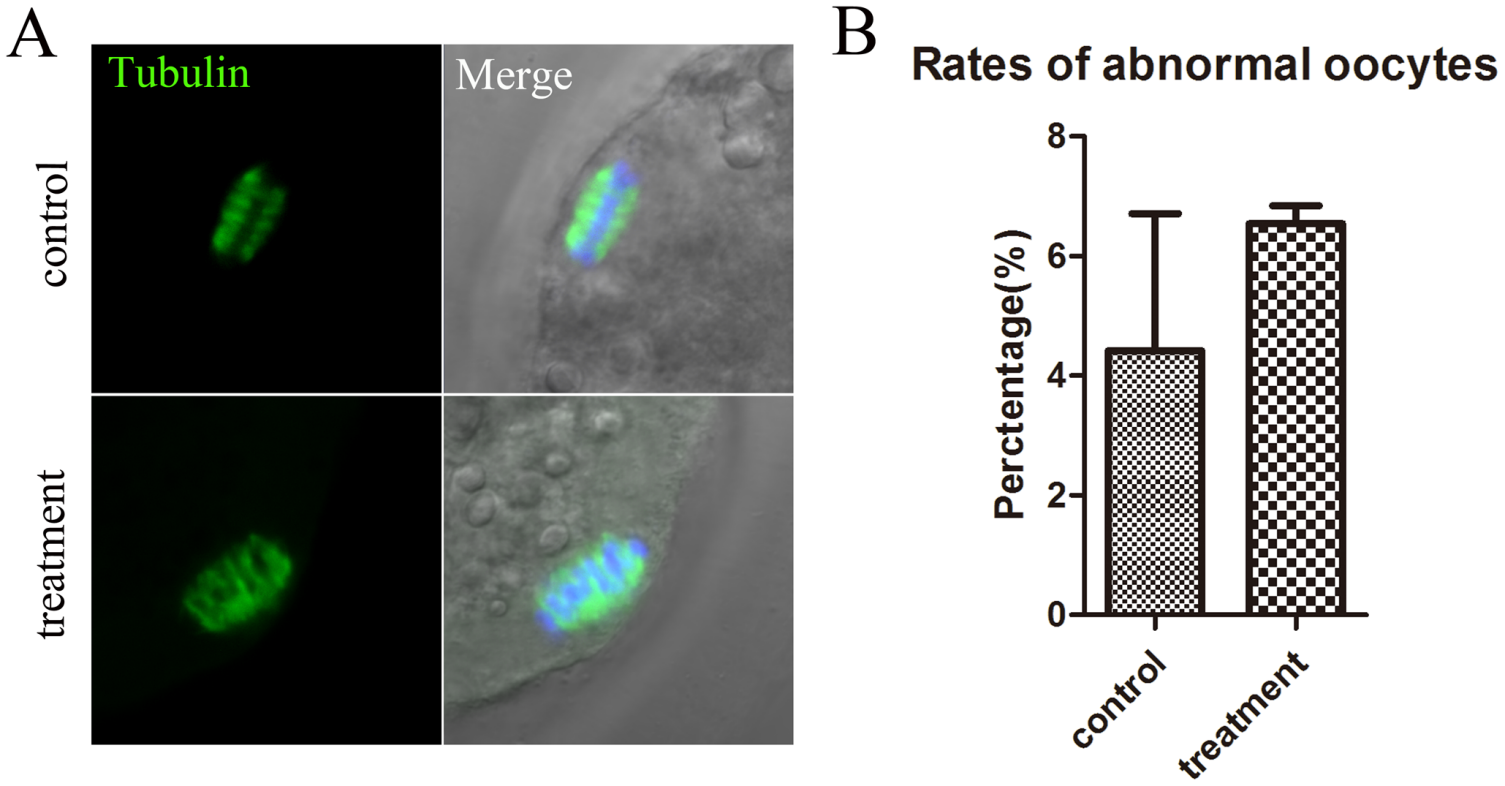
Effects of KIF20A inhibition on spindle structure and chromosome alignment of porcine oocytes. (A) Normal Spindle structure and chromosome alignment were investigated after KIF20A inhibition. (**B**) Rates of abnormal oocytes (displaying chromosome misalignment and aberrant spindles) after KIF20A inhibition.

To further explore the reason for the failure of porcine oocyte maturation, we also examined spindle formation after KIF20A inhibition. As shown in Figure 3A, there is no difference between the control and treatment groups for spindle morphology. In the control group, 4.42±2.29% (n = 45) of oocytes showed abnormal spindle morphology compared with 6.55±0.30% (n = 46) of 50 µM treated oocytes (p>0.05) (Fig. 3B).

### Intracellular localization of KIF20A during porcine early embryo development

**Figure 4:**
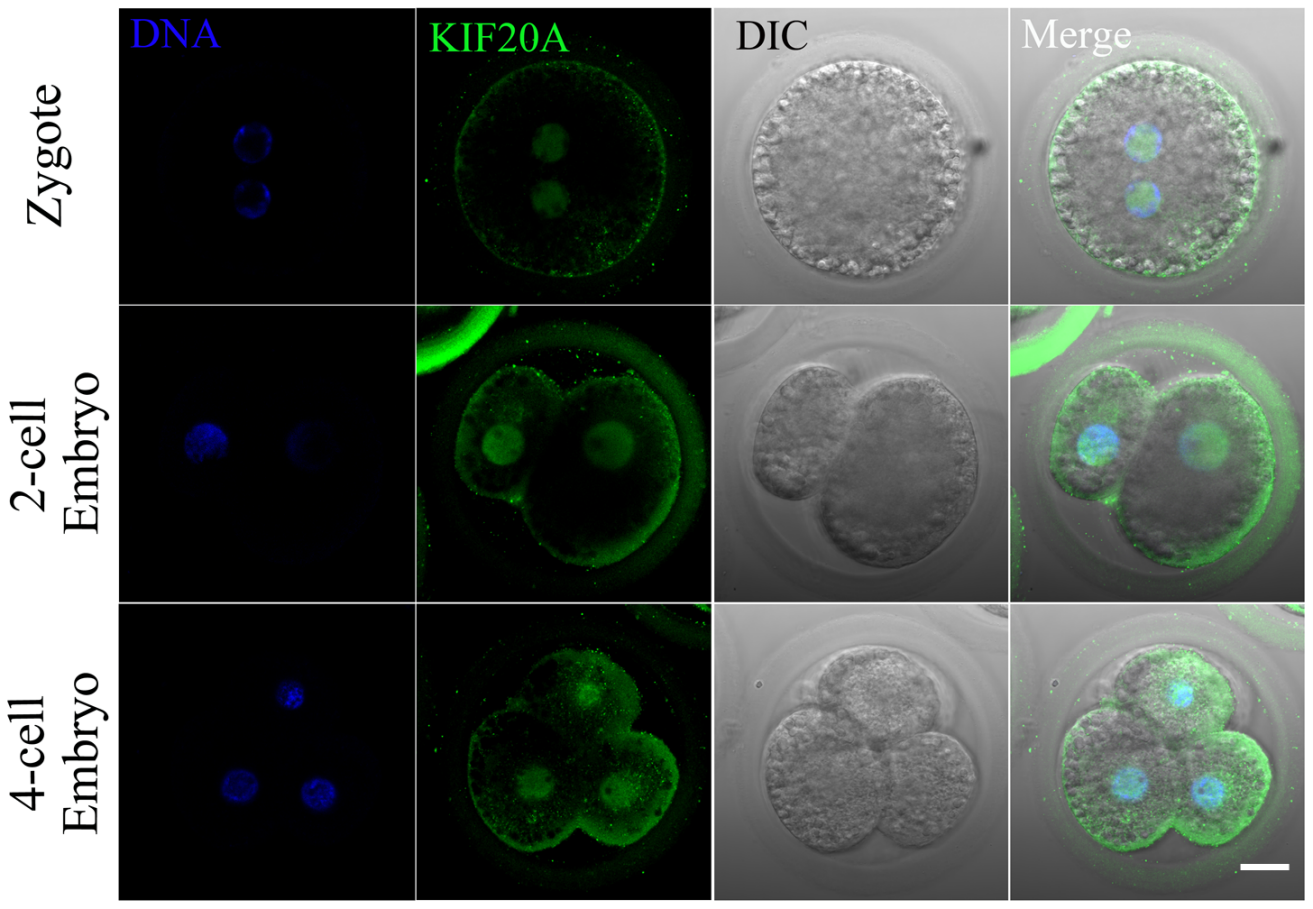
Localization of KIF20A in porcine early embryo. Samples were collected from zygote the to the 4-cell stage. KIF20A was primarily accumulated at the nucleus from the zygote to the 4-cell embryos stages. Green, KIF20A; blue, chromatin. Bar  = 20 µm.

The subcellular localization of KIF20A at different developmental stages of porcine early embryos was examined. After culturing for 24 or 48 h when the activated oocytes should develop to 2-cell or 4-cell stage, early embryos were examined using immunofluorenscent staining. As shown in Figure 4, KIF20A was primarily localized in the nucleus from the zygote to the 4-cell stage.

### Inhibition of KIF20A activity results in the arrest of early embryo development

**Figure 5:**
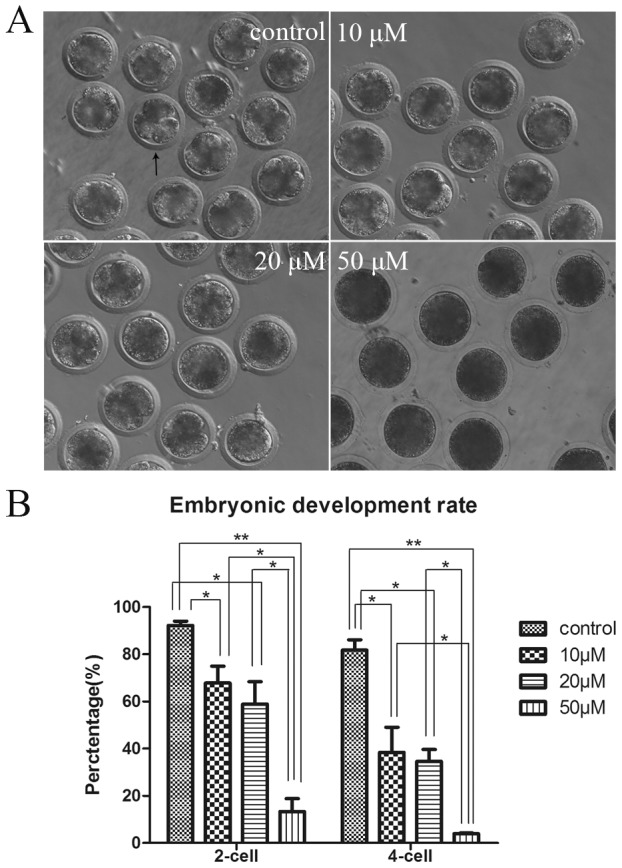
KIF20A inhibition affects the divisions of embryos. (A) Schematic diagram of cleavage of early embryos in different groups. The black arrow indicated a 4-cell embryo in control group.(B) Effect of KIF20A inhibition on cleavage ability of zygotes. The embryonic development rate has dropped after KIF20A activity inhibition. Almost all of the embryos cleaved normally in the control groups, while the cleavage of embryos was blocked in treatment groups, especially in the 50 µM groups. * significant difference (p<0.05).

To investigate the role of KIF20A in embryo development, embryos were treated with Paprotrain from the zygote stage to inhibit endogenous KIF20A activity during embryo development after EST. As shown in Figure 5A, most embryos underwent cleavage in the control groups, while those treated were blocked at the zygote or 2-cell stage, especially when treated with 50 µM inhibitor. Figure 5B shows that 92.15%±1.83% (n = 115) of MII oocytes in control groups developed to the 2-cell stage, while only 67.77%±12.02% (n = 91; p<0.05) of 10 µM KIF20A inhibitor-treated MII oocytes, 58.83%±13.41% (n = 119; p<0.05) of 20 µM KIF20A inhibitor-treated MII oocytes and 13.30%±6.59% (n = 112; p<0.01) of 50 µM KIF20A inhibitor-treated MII oocytes developed to the 2-cell stage. After culturing for 48 h, 81.73%±3.80% (n = 115) of MII oocytes in control developed to 4-cell stage, whereas the developmental rate dropped to 38.42%±16.34% (n = 91; p<0.05), 34.55%±8.64% (n = 119; p<0.05) and3.91%±1.36% (n = 112; p<0.01) for 10, 20, 50 µM KIF20A inhibitor-treated groups, respectively. These results showed that inhibition of KIF20A activity resulted in failure of early embryo development in a dose-dependent manner.

## Discussion

The specific inhibitor, Paprotrain, was used in our study to investigate the possible function of KIF20A during pig oocyte meiotic maturation and early parthenogenetic embryo development. The results showed that KIF20A significantly affects porcine oocyte maturation and early embryo development. Whether or not KIF20A is expressed in porcine oocytes was also first examined. These results confirmed that KIF20A had a specific dynamic localization during porcine oocyte maturation. Our results showed that KIF20A localized at the centromeres of either MI or MII stages and was restricted to the midbody at ATI stage, which is similar to a previous report that KIF20A co-localized with Aurora B at the central spindle in AI and overlapped with Aurora B at the midbody in TI [18]. In addition, the localization was consistent with the result that KIF20A localized at the central mitotic spindle at TI, which is required for completion of cytokinesis during mitosis [21]. Therefore, KIF20A could possibly perform function during porcine oocyte meiotic maturation.

To verify the above hypothesis, the activity of KIF20A was inhibited by Paprotrain. Result showed that polar body extrusion by porcine oocytes was inhibited as well, indicating a failure of meiotic maturation in porcine oocytes. Recent reports indicate that components concerned with the central spindle are individually involved with cytokinesis in most animal cells, and multiple mitotic kinesins acts on cytokinesis directly [22]. KIF14 as well as KIF4 have an essential function in cytokinesis through interacting with the central spindle [23], [24]. Some studies indicate that MKLP1 is essential for cytokinesis [25], [26]. It has been reported that KIF20A is also involved in cytokinesis [10], via affecting the localization of a subset of central spindle components, including PLK1 [11]. Recently, inhibition of KIF20A results in a cytokinesis defect and the production of binucleated cells in HeLa cells [27]. Therefore, it is considered that KIF20A is crucial for meiosis of porcine oocytes.

In addition, we further explored the mechanism by which KIF20A inhibitor affected polar body extrusion. Recently, results from several studies suggested that KIF20A participated in regulating the localization of chromosomal passenger complex (CPC), a positive regulator of cell cycle progression, from centromeres to the central spindle during transition of the MI to AI [10], [18]. Drosophila Subito, homologous with KIF20A, is also required for the localization of the CPC [19], [28]. CPC regulates the orientation of homologous chromosomes during meiotic cell division [29], and CPC knockdown results in cell cycle progression defects in both mouse and Caenorhabditis elegans oocytes [30]–[33]. The failure of the relocation of CPC to the central spindle also results in the failure of cell cycle progression. Furthermore, KIF20A displays a cell cycle–regulated expression pattern, and is crucial for late anaphase B and/or cytokinesis [34]. Previous studies confirmed that depletion of KIF20A by RNAi resulted in cytokinesis defects, leading to an increased binucleated cells [10], [17]. Our study, found that inhibition of KIF20A activity caused the disturbance of cell cycle progression; most oocytes arrested at early MI stage after KIF20A inhibition. This is similar with the previous reports. Moreover, our results also showed that the involvement of KIF20A on oocyte maturation is not dependent upon effects on the spindle, since most oocytes showed normal spindle morphology after inhibitor treatment. Taken together, these results suggest that KIF20A might be important for accomplishment of meiotic maturation by regulating of cell cycle progression rather than spindle assembly.

In addition, we also investigated the function of KIF20A in porcine embryos. The expression of KIF20A during different stages of early embryo development was detected. Our results showed that KIF20A accumulated in the nucleus of porcine early embryos during all developmental stages studied, which was consistent with a previous report that KIF20A accumulated in the nucleus during the G2 phase of the cell cycle in human primary hepatocytes [35]. Therefore, KIF20A might play a significant role during porcine embryo development. To verify the function of KIF20A during embryo development, we disrupted its activity by Paprotrain treatment during the zygote stage. Previous results showed that Zen-4, which belongs to the same family of kinesin 6 proteins and homologous with MKLP1, is required for embryogenesis [25]. In addition KIF20A knockdown inhibited cell proliferation in hepatoma cells. Our research showed that cleavage of early embryos was disturbed after inhibition of KIF20A, especially in the 50 µM KIF20A inhibitor-treated groups. Therefore, KIF20A might contribute to the regulation of porcine early embryos development.

In summary, our results indicated that KIF20A did regulate porcine oocyte meiotic maturation and early embryo development. Clearly, further research is in need to elucidate the underlying mechanism of KIF20A on pig oocyte meiosis.
